# Adaptive Resource Optimization for LoRa-Enabled LEO Satellite IoT System in High-Dynamic Environments

**DOI:** 10.3390/s25113318

**Published:** 2025-05-25

**Authors:** Chen Zhang, Haoyou Peng, Yonghua Ji, Tao Hong, Gengxin Zhang

**Affiliations:** College of Telecommunications and Information Engineering, Nanjing University of Posts and Telecommunications, Nanjing 210003, China

**Keywords:** LEO satellite, IoT, LoRa, spreading factor, resource optimization

## Abstract

The integration of Low-Earth Orbit (LEO) satellites with Long Range Radio (LoRa)-based Internet of Things (IoT) systems for extensive wide-area coverage has gained traction in academia and industry, challenging traditional terrestrial resource optimization designed for semi-static single-base-station environments. This paper addresses LEO’s high dynamics and satellite-ground channel variability by introducing a beacon-triggered framework for LoRa-LEO IoT systems as a foundation for resource optimization. Then, in order to decouple the intertwined objectives of optimizing energy efficiency and maximizing the data extraction rate, an adaptive spreading factor (SF) allocation algorithm is proposed to mitigate collisions and resource waste, followed by a practical dynamic power control mechanism optimizing LoRa device power usage. Simulations validate that the proposed adaptive resource optimization outperforms conventional methods in dynamic, resource-constrained LEO environments, offering a robust solution for satellite IoT applications. In terms of energy efficiency and data extraction rate, the algorithm proposed in this paper outperforms other comparative algorithms. When the number of users reaches 3000, the energy efficiency is improved by at least 119%, and the data extraction rate is increased by at least 48%.

## 1. Introduction

### 1.1. Background and Contribution

In recent years, the ongoing advancement and widespread adoption of Internet of Things (IoT) technology have led to the deployment of an increasing number of devices and sensors across diverse environments, resulting in the establishment of extensive IoT systems. However, existing terrestrial networks cannot meet the demands of various application scenarios, particularly in remote areas or complex environments where the costs associated with the construction and maintenance of terrestrial infrastructure can be prohibitively high. For regions lacking terrestrial network access, such as oceans, deserts, and forests, Low-Earth Orbit (LEO) satellite IoT can be utilized for comprehensive data transmission, thereby addressing the challenges of IoT coverage and connectivity [1].

To fulfill the requirements for low power consumption and long-distance transmission, Low Power Wide Area Network (LPWAN) technology is particularly suitable for LEO satellite IoT. Among the available LPWAN technologies, Long Range Radio (LoRa) has garnered significant attention due to its ability to be directly deployed on public networks, low cost, and the absence of a requirement for specific frequency band usage authorization [2].

In the industrial field, numerous examples of LoRa technology implemented within LEO satellite IoT can be found, including initiatives from companies such as Lacuna Space and Tianqi. Lacuna Space is a UK startup that provides low-power IoT connectivity using LEO satellites and CubeSats with LoRaWAN technology to connect devices globally. The Tianqi satellites are China’s first LEO constellation for IoT data transmission, consisting of 38 satellites. Several have been launched, forming a global real-time satellite IoT communication system. They uniquely use LoRa technology for seamless connectivity between ground IoT devices and space stations.

However, despite the application of LoRa technology for LEO satellite IoT in the industrial sector, its construction and operation encounter various challenges, particularly regarding resource optimization. Satellite communication systems operate under resource constraints, and the increasing number of IoT terminals, along with the growing complexity of scenarios, exacerbates the scarcity of wireless resources in LEO satellite IoT, significantly impeding its advancement. Therefore, the rational allocation and scheduling of resources emerge as critical strategies to mitigate this issue [1].

The main problems regarding LEO satellite IoT and LoRa technology application are as follows: LoRa application faces scarce wireless resources due to satellite communication constraints and growing IoT complexity. Traditional methods fail to adapt to LEO’s dynamic scenarios, and the intertwined goals of optimizing energy efficiency and data extraction rate for satellite IoT terminals with long distance transmission need to be decoupled through proper algorithms and power control.

To address these above issues, this paper thoroughly takes into account the high dynamics of LEO, the satellite-ground channels, provides a beacon-triggered framework for LoRa-Enabled LEO satellite IoT System, and serves as the foundation for resource optimization. Then, in order to decouple the intertwined objectives of optimizing energy efficiency and maximizing the data extraction rate, a two-step solution approach is adopted. A novel SF allocation and transfer algorithm is proposed to mitigate collisions and minimize resource waste.

Extensive simulation results show that the proposed adaptive resource optimization algorithm can allocate resources adaptively and significantly outperforms multiple conventional methods. This enhancement makes the proposed algorithm particularly suitable for the dynamic and resource-constrained environments of LEO satellites, offering a robust solution for satellite IoT applications.

The main contributions of this paper are as follows:

1. This study introduces a novel beacon-triggered framework for LoRa-enabled LEO satellite IoT systems, which includes modifications to the calculation formulas for frame duration and skip probability. Unlike conventional solutions, the proposed architecture explicitly addresses the fundamental disparities between semi-static terrestrial networks and highly dynamic LEO scenarios, where orbital motion-induced channel conditions and received signal power variations significantly impact link reliability. This framework serves as the foundation for subsequent resource optimization algorithms, marking a paradigm shift from traditional semi-static approaches to fully adaptive, space-aware IoT resource management.
2. Building upon the beacon-triggered framework, this paper explicitly incorporates the characteristics of on-board satellite processing to analyze the conditions for successful data extraction. By accounting for the energy constraints of satellite IoT terminals and their significantly longer transmission distances compared to terrestrial scenarios, the objective function integrating energy efficiency and data extraction rate is formulated.
3. To decouple the intertwined objectives of optimizing energy efficiency and maximizing data extraction rates, an adaptive SF allocation algorithm is proposed. This algorithm aims to mitigate collisions and minimize resource waste. Complementing this, a practical dynamic power control mechanism is introduced to optimize power usage of LoRa devices. Together, these strategies enhance overall system performance by effectively balancing energy efficiency with data throughput.

### 1.2. Related Works

In academic research, efforts have been made by scholars focusing on resource optimization for terrestrial IoT utilizing LoRa communication technology. The work of [3] proposes a method that enables terminals to adaptively select parameters such as bandwidth and spreading factor (SF) based on the relationship between received power and sensitivity, thereby minimizing data packet airtime and optimizing power to reduce overall system energy consumption. The work of [4] introduces a novel adaptive data rate algorithm for LoRaWAN networks, aiming to enhance network performance by reducing packet collisions in the LoRaWAN environment. The work of [5] integrates the cross-layer design concept of resource sharing, proposing an SF allocation method based on the LoRaWAN network that considers path loss constraints and a model relating to transmission success rates. Additionally, the work of [6] introduces the EXPLoRa algorithm for effective wireless resource distribution, which averages SF allocation among terminals based on the total number of devices connected within the gateway coverage area and constraints of received signal strength, ultimately improving system reliability and throughput. The aforementioned LoRa-based resource optimization algorithms predominantly focus on terrestrial scenarios, targeting static IoT environments.

In the context of satellite IoT, scholars have made considerable efforts in studying the applications of satellite IoT. The work of [7] proposed a solution for monitoring athletes in extreme environments based on satellite IoT. The work of [8] proposed a sparse satellite constellation design for global and regional direct-to-satellite Internet of Things services. The work of [9] studied the characteristics of mission-critical applications, the motivation for the integration of satellites and the IoT.In the context of LoRa satellite IoT, the work of [10] proposes a scheduling algorithm for LoRa terminals communicating with LEO satellites. This algorithm departs from traditional ALOHA-based LoRa approaches and employs a Time Division Multiple Access (TDMA) method to ensure reliable communication links, thereby mitigating packet loss and collisions. This strategy significantly enhances the performance of terminals. The work of [11] introduces a scalable and energy-efficient downlink to satellite Internet of Things (DtS-IoT) method based on LoRa technology. This approach employs a Mixed Integer Linear Programming (MILP) model to implement uplink transmission strategies in accordance with satellite trajectories. However, this scheduling strategy necessitates intricate transmission scheduling calculations for each device, rendering it impractical for real-world applications due to the extensive information required. Consequently, it functions primarily as a theoretical upper limit reference for optimizing channel utilization. To tackle the challenging issue of efficiently allocating SF to terminal devices in large-scale non-terrestrial network LoRaWAN and minimizing co-SF interference, the work of [12] investigates an SF allocation scheme based on reinforcement learning aimed at optimizing the system’s energy efficiency.

While previous studies have made valuable progress in LoRa-based resource optimization for satellite IoT, they still face significant challenges. Existing works, such as [10,11,12], have explored various scheduling algorithms and resource allocation methods, aiming to enhance the performance of satellite IoT systems. For instance, [10] proposed a TDMA-based scheduling algorithm to improve communication reliability, [11] introduced an energy-efficient DtS-IoT method based on MILP model, and [12] investigated an SF allocation scheme using reinforcement learning. 

However, there are notable differences between our work and these previous studies. Most existing terrestrial LoRa scheduling algorithms, like those mentioned above, mainly adopt Class A mode with fixed resource configurations. This approach fails to adapt to the dynamic channel changes caused by the high-speed movement of satellites, a limitation that our research seeks to overcome. Regarding energy resources, previous studies often use fixed power configurations, which cannot achieve optimal energy efficiency and may lead to resource waste, while our work focuses on developing more flexible and energy-efficient power management strategies. Moreover, unlike previous research that has not fully utilized the orthogonality of SF in the LoRaWAN system to improve resource utilization, our study delves deep into leveraging this orthogonality to optimize resource allocation, thereby enhancing the overall performance of the satellite IoT system [1,2].

The rest of the paper is organized as follows: Section 2 presents the system model of LEO Satellite IoT based on Lora. The resource optimization algorithm is described in Section 3. Section 4 provides Simulation and Analysis. Future work and Conclusions are discussed in Section 5 and Section 6.

## 2. System Model

### 2.1. LEO Satellite IoT Based on LoRa

The LoRa technology, recognized as a low-power solution for the IoT, is distinguished by its low energy consumption, extensive transmission range, robust anti-interference capabilities, and high sensitivity in receiving signals [13]. These attributes render it particularly suitable for applications within LEO satellite IoT environments. In such a system, IoT terminals located within the satellite’s coverage area transmit data packets to the LEO satellites through an uplink. The satellite receiver then demodulates these packets, and the resulting information is relayed via a feeder link to a ground gateway, which connects to the core network to facilitate IoT services. Throughout this process, resources such as transmission power and SFs for ground terminals are managed and allocated by the satellite. The SF of LoRa ranges from 7 to 12. The key feature of the SF is its orthogonality, which allows multiple signals with different SFs to coexist in the same frequency band without interference.

However, the LEO satellite LoRa system differs significantly from traditional terrestrial LoRa systems. Notable distinctions exist in network architecture, channel characteristics, and resource optimization.

Firstly, in terms of network architecture, the terrestrial LoRa system typically relies on a communication model involving fixed base stations and static terminals. Ground-based base stations have limited coverage, with terminal devices communicating through single-hop or multi-hop connections. This architecture is relatively straightforward, characterized by fixed base station locations and limited terminal mobility. In contrast, the LEO satellite LoRa system employs a communication model between satellites and ground terminals. The satellites orbit at high speeds, offering extensive coverage, while terminal devices communicate over long distances via these satellites, leading to a more complex network architecture in which terminals may transition between different satellites [14].

Secondly, concerning channel characteristics, the terrestrial LoRa system experiences relatively stable channel conditions, primarily influenced by terrain, buildings, and weather. While multipath effects and small-scale fading present challenges, the slow variability of the channel allows for optimization based on a fixed channel model. In contrast, the channel conditions in the LEO satellite LoRa system are subject to rapid changes due to the high-speed motion of satellites and free space loss. The varying distance and elevation angle between the satellite and terminal result in swift fluctuations in received power and channel conditions, necessitating real-time adaptation of resource optimization strategies.

**Figure 1:**
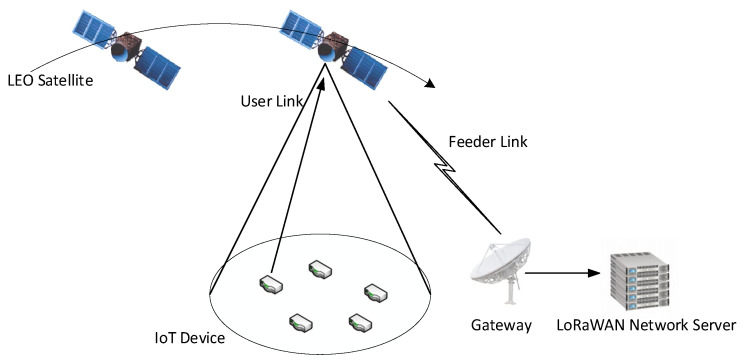
LEO satellite LoRa system model.

Finally, regarding resource optimization, the terrestrial LoRa system typically allocates resources based on fixed SF and transmission power configurations [15]. The scheduling period is relatively long, ranging from several minutes to hours, and resource allocation is straightforward, primarily focusing on terminal signal strength and service priority. Conversely, resource optimization in the LEO satellite LoRa system requires real-time adjustments to accommodate the satellites’ high-speed movement and dynamic channel variations. This results in shorter scheduling periods and more complex resource allocation processes, which involve dynamic modifications of SFs and transmission power. By accounting for the dynamic factors associated with satellite movement, effective integration and scheduling of onboard resources under constrained conditions can facilitate enhanced management and optimized connectivity for IoT devices. This is essential for improving the overall performance of satellite IoT [16]. The LEO Satellite LoRa system model is shown in Figure 1.

The channel model utilized in this study incorporates considerations of attenuation and interference, distinguishing it from conventional terrestrial static IoT systems. Given that the IoT devices discussed herein are serviced by LEO satellites, these devices typically feature antennas and transmission capabilities that surpass those of standard terrestrial IoT devices. Additionally, the communication links of these devices primarily rely on line-of-sight paths, allowing for the partial neglect of small-scale fading effects.

As the satellite moves, it impacts the received signal at the satellite. Consequently, to ensure the successful reception of data packets, each user must employ distinct modulation parameters and transmission power levels. The received power at the satellite can be calculated using the following formula [17]:

(1) $$P_{r}=EIPR+G_{r}-FSPL-A_{\mathrm{loss}}-Ad_{\mathrm{loss}},$$

where EIPR is the equivalent isotropic radiated power of the transmitting antenna, $G_{r}$ is the antenna gain, FSPL is the free space propagation loss, $A_{\mathrm{loss}}$ and $Ad_{\mathrm{loss}}$ represent atmospheric losses due to gases, rain, etc., and additional losses caused by the feedline, respectively.

To ensure the successful demodulation of the data packet, the following condition must be met [11]:

(2) $$P_{r}\geq S_{SF},\;S_{SF}=10\mathrm{lg}(kT\times 10^{3})+10\mathrm{lg}(BW)+NF+SNR_{SF},$$

where k is the Boltzmann constant, T is the temperature, BW is the bandwidth, NF is the noise figure, $SNR_{SF}$ is the signal-to-noise ratio, $S_{SF}$ is the sensitivity. If the received power is greater than or equal to the sensitivity value, the receiver will be able to receive the data packet.

With respect to the Doppler shift issue, the studies [18,19] implemented Doppler shift compensation techniques to mitigate the adverse effects of Doppler shift on LoRa communication systems at the physical layer. Given that the primary focus of this paper lies in the Medium Access Control (MAC) layer, in depth discussions on physical layer designs will not be elaborated upon herein.

### 2.2. Beacon Mechanism of LEO Satellite IoT Based on LoRa

**Figure 2:**
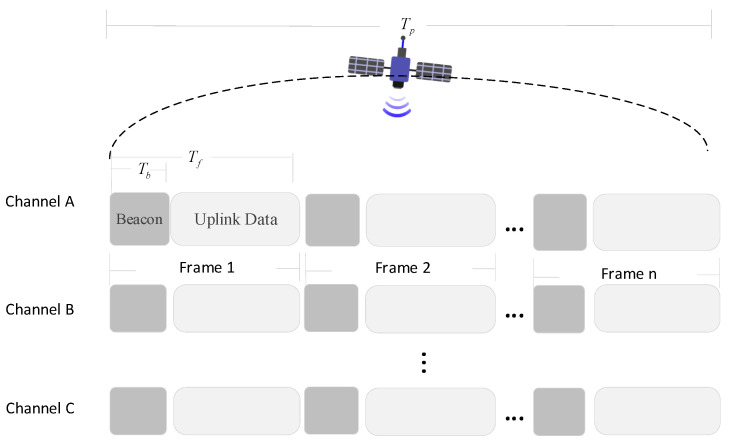
Data frame structure model triggered by the beacon.

The LEO satellite’s dynamic movement across the area of IoT devices creates a highly variable channel, requiring the development of tailored media access schemes. Furthermore, network operations must minimize costly handshakes and conserve power when the satellite is not in view. These objectives can be achieved through a beaconing approach similar to that of LoRaWAN Class B [20]. Inspired by the work of [11], a data frame structure model activated by beacons has been proposed, which modified the calculation formulas for frame duration and skip probability. The data frame structure model is shown in Figure 2.

The uplink transmissions from devices are organized into frames, which are triggered by beacons sent periodically by the LEO satellite. The satellite utilizes the most robust SF to send these beacons, which users can receive and decode while within the satellite’s line of sight. Since SF12 is the most robust SF, it offers the maximum communication range and the strongest anti-interference capability, ensuring that devices can receive the beacons even under the worst channel. Furthermore, if a device can successfully receive and decode the SF12 beacon, it can proceed with its uplink transmission.

After successfully demodulating the beacon, users are allowed to send data to the satellite. Depending on factors such as policies and channel conditions, users can transmit during the satellite’s passage using specific configurations within designated channels and frames, specifically with particular SFs and power levels [11].

(3) $$\sum_{sf}\sum_{c}p_{sf,c}^{i,b}\leq 1,\forall i,\;b.$$

Each user must ensure that the number of data packets sent during the satellite’s passage does not exceed the number of packets stored in the local cache, which is

(4) $$\sum_{b}\sum_{sf}\sum_{c}p_{sf,c}^{i,b}\leq m,\forall i.$$

Simultaneously, each data packet can exist in only one channel at most.

(5) $$\sum_{c}p_{sf,c}^{i,b}\leq 1,\forall sf,i,b.$$

The satellite passes through time $T_{p}$, which is divided into n slots. Each slot has a duration $T_{f}$, which includes the signaling time $T_{b}$. To ensure that the satellite can complete the transmission of cached data during the time it passes through, the frame duration $T_{f}$ must satisfy the following conditions [11]:

(6) $$T_{b}\leq T_{f}\leq \frac{T_{p}}{n},$$

where n indicates the total number of slots.

On the other hand, based on the signaling structure model for the transmission, the transmitted signal contains important information regarding the expected number of users N served by the system. The terminal equipment can determine whether to transmit in the current slot or skip to the next slot based on the output of the skip function defined in (9):

(7) $$P_{skip}=\frac{2}{1+\exp\left(-\frac{N}{p_{\mathrm{skip}}}\right)}-1,$$

where $p_{\mathrm{skip}}$ is a parameter that indicates the number of skipped slots (depending on the agreement and the number of channels, etc.), and $P_{skip}$ is the probability of skipping the current slot. Specifically, as $p_{\mathrm{skip}}$ increases, the likelihood of skipping a slot also increases, resulting in a higher probability of occurrence.

### 2.3. The Optimal Distribution of SF

Based on the ALOHA data transmission model [21], the SF is introduced as a new variable. In this model, user requirements dictate that the SF should be able to adjust between low and high values effectively. When the system contains a certain SF, arbitrary connections can be established to obtain the opportunity for data packets generated by this SF in a single collision instance, denoted as $p_{coll,SF}$, as expressed:

(8) $$p_{coll,SF}=1-e^{-2G_{SF}},SF\in \{7,8,9\ldots \ldots ,12\},$$

where $G_{SF}$ represents the volume of data packets generated in the network with the same SF.

The transmission time of the data packet $T_{s}$ and the effective data length are defined, as shown in (9) and (10):

(9) $$T_{s}=PL/R_{b},$$

and

(10) $$R_{b}=\frac{SF\times BW\times CR}{2^{SF}},\;SF\in \{7,8,9,10,11,12\},$$

where PL denotes the effective length of the data packet, and $R_{b}$ represents the transmission rate.

The number of data packets λ generated in the network per unit time is expressed as shown in (11):

(11) λ=N/T,

where N denotes the total number of terminals within the network, and T represents the average time interval between the arrivals of data packets.

Combining (8)–(11), and incorporating the proportion of the user’s selection of the SF [21], as shown in (12):

(12) $$G_{SF}=\lambda T_{s}P_{SF}=\frac{2^{SF}}{SF}\frac{PL\times N}{BW\times CR\times T}P_{SF},SF\in \{7,8,9\ldots \ldots ,12\}.$$

Thus, the probability of successful transmission using the SF in the LoRaWAN network can be expressed as:

(13) $$p_{coll,SF}=1-e^{-\left[\frac{2^{SF+1}}{SF}\frac{PL\times N}{BW\times CR\times T}P_{SF}\right]},SF\in \{7,8,9\ldots \ldots ,12\}.$$

To minimize collision probabilities, the following equation needs to be solved:

(14) $$\min\max p_{coll,SF}.$$

The collision probabilities are minimized when and only when the following equation holds. The equation is as follows:

(15) $$\begin{array}{l} p_{coll,SF=i}=p_{coll,SF=j}\\ i,j\in \{7,8\ldots 12\},i\neq j \end{array}.$$

When the probability of successful transmission at different SF is minimized, the distribution of the SFs is optimized based on the maximum network performance achievable during the specified time [19]. This is represented as follows:

(16) $$e^{-\left[\frac{2^{i+1}}{i}\frac{PL\times N}{BW\times CR\times T}P_{i}\right]}=e^{-\left[\frac{2^{j+1}}{j}\frac{PL\times N}{BW\times CR\times T}P_{j}\right]},i,j\in \{7,8\ldots 12\},i\neq j,$$

where $P_{i}$ and $P_{j}$ represent the proportions of i and j.

The proportion relationship between i and j can be expressed as

(17) $$P_{i}=\frac{2^{j+1}}{j}P_{j}\times \frac{i}{2^{i+1}},i,j\in \{7,8\ldots 12\},i\neq j.$$

The proportions of SFs across all nodes in the network are constant. This is represented as follows:

(18) $$\sum_{i=7}^{12}P_{i}=1.$$

From Equations (17) and (18), the optimal distribution of the SF can be derived, as shown in Equation (19):

(19) $$P_{SF}=\frac{SF}{2^{SF}}/\sum_{i=7}^{12}\frac{i}{2^{i}}.$$

The above distribution applies to general scenarios. In scenarios with high real-time data requirements such as disaster detection, optimization for the age of information (AoI) is necessary [22,23].

In this paper, considering disaster detection and other scenarios with high requirements for real-time data, the adaptive adjustment mechanism is added. In disaster monitoring scenarios, the system gives priority to guaranteeing the transmission of critical data. At the same time, the SF is dynamically adjusted to assign smaller SFs to users in the disaster monitoring scenario as much as possible to meet their requirements for low information age, thus speeding up the transmission of critical data and ensuring that data can be delivered quickly and accurately. The AoI is defined, as shown in Equation (20):

(20) $$\mathrm{AoI}=\frac{PL\times 2^{SF}}{SF\times BW\times CR},\;SF\in \{7,8,9,10,11,12\}.$$

As shown in Equation (20), low SF helps to deliver critical data to the receiving end as soon as possible, so that the receiving end can obtain the latest information in time to make the corresponding decisions.

## 3. Adaptive Resource Optimization

### 3.1. Optimization Problem

It is crucial to thoroughly consider both the data retrieval requirements and the energy-saving needs of IoT terminals. Within the designated range of SFs and power levels, a resource optimization algorithm based on the LoRa mechanism is developed to maximize the system’s energy efficiency and data retrieval rate.

The energy efficiency (EE) of the system is defined as the amount of successfully retrieved data per unit of power consumed, as expressed by the following Equation:

(21) $$EE=\sum_{i=1}^{N}EXT_{i}\cdot PL/\sum_{b}p_{i}^{b}*TOA_{i}^{b},$$

where $EXT_{i}$ represents the number of data packets successfully retrieved by user i from the satellite network. It is related to the SF and the transmission power selected. PL represents the length of the data packet. $p_{i}^{b}$ represents the transmission power of user i within the slot b, and $TOA_{i}^{b}$ represents the duration for which user i transmits data packets in this slot. It is directly related to the SF selected by the user; if user i does not transmit in slot b, then $p_{i}^{b}$ is considered to be zero.

The Data Extraction Rate (DER) of the system is defined as the ratio of successfully retrieved data packets to the total data packets. The data extraction rate reflects, to some extent, the impact of the selected SF on the system’s transmission success rate. The DER is represented as:

(22) $$DER=\frac{\sum_{i=1}^{N}EXT_{i}}{Nm},$$

where $N_{m}$ represents the number of total data packets.

Energy efficiency partially reflects the resource allocation of SFs and power within the system, while the data extraction rate more accurately represents the SF configuration. Additionally, there is a relationship between the system’s data extraction rate and its energy efficiency; specifically, energy efficiency tends to increase as the data extraction rate rises. However, it is important to note that both metrics do not reach their maximum values simultaneously. Therefore, the following optimization problem is proposed:

(23) $$\max[EE(SF_{i}^{b},p_{i}^{b}),DER(SF_{i}^{b},p_{i}^{b})],$$

(24) $$C_{1}:7\leq SF_{i}^{b}\leq 12,$$

(25) $$C_{2}:p_{\min}\leq p_{i}^{b}\leq p_{\max},$$

(26) $$C_{3}:P_{r}\geq S_{SF},$$

(27) $$C_{4}:N_{arrival}\leq L,$$

(28) C5:ifSFi=SFj or tn=tm packeti∩packetj=∅.

Specifically, the constraint C1 indicates the range of SF for LoRa. The constraint C2 specifies the power control range. The constraint C3 indicates that the received power is greater than the sensitivity threshold, ensuring no packet loss. The constraint C4 indicates that the number of arriving packets is less than the number of packets that the satellite can process simultaneously, ensuring no unprocessed packets. The constraint C5 indicates no packet collisions.

### 3.2. Resource Optimization Strategy

To enhance the energy efficiency and data extraction rate of LEO satellite IoT systems, the constraints of this optimization problem include operating within the specified range of SFs and designated power control limits, while also ensuring the successful retrieval of data packets. In LEO satellite IoT scenarios, the rapid changes in channel conditions due to the satellite’s high-speed movement necessitate frequent recalculations of the optimization results, which significantly increases the complexity of the optimization problem.

**Figure 3:**
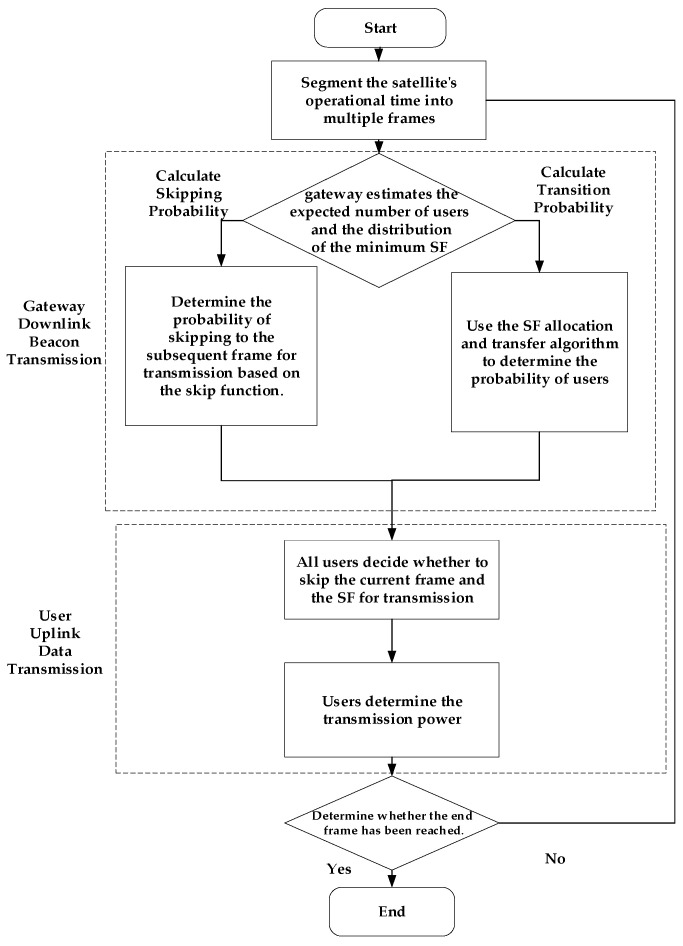
Framework of the LoRa-based LEO satellite IoT resource optimization algorithm.

The selection of SF is intricately linked to transmission power, thereby complicating the optimization process within LEO satellite IoT systems. To tackle this challenge, a decoupling approach for SF and transmission power is essential. In traditional ground-based LoRa IoT scheduling, SF and power control calculations are typically conducted simultaneously, under the assumption of stable base station positions and minimal fluctuations in channel conditions. However, in the context of LoRa-based LEO satellite IoT, the high-velocity movement of LEO satellites induces rapid variations in distance and channel conditions, rendering simultaneous calculations of SF and power control highly challenging. This concurrent computation significantly amplifies complexity, posing substantial implementation hurdles for typical IoT devices. To address this bottleneck, the paper first establishes the transition probabilities of the SFs and transmits these probabilities to users. Users can then derive their respective SFs based on these probabilities and leverage them to regulate their transmission power. This study proposes an energy-efficient LoRa scheduling strategy tailored for LEO satellite IoT, prioritizing SF selection at the satellite. Subsequently, users adjust their transmission power according to the selected SF results. The overall framework is visually depicted in Figure 3.

The algorithm operates as follows: The LEO satellite service time is divided into several frames based on the beacon-triggered data frame structure model. In each frame, downlink beacon transmission occurs first, followed by uplink data transmission. Before the downlink beacon transmission, the gateway assesses the expected number of service users within that frame and the distribution of minimum SF ratios based on historical data. The skip function in Equation (7) is then used to determine the probability of data packets skipping to subsequent frames for transmission. Simultaneously, a target SF ratio distribution is established using the SF allocation algorithm, and the probabilities of all SFs transitioning to others are determined via the SF transfer algorithm. This probability data and synchronization information are included in the beacon transmission.

During the uplink data transmission phase, users determine their SFs based on the transition probabilities received in the beacon and decide whether to transmit in the current frame and which specific SF to use according to the beacon information. Users then adjust their transmission power based on their individual conditions. This process continues until all frames have been iterated through, at which point the algorithm concludes.

It is important to note that for ground-based LoRa user terminals, the transmission power typically ranges from −4 dBm to 20 dBm [3]. However, due to the greater distance to LEO satellites compared to ground-based LoRa terminals, the transmission power range often needs to be increased. Therefore, effective power control in the LoRa system can help manage user energy consumption, enhancing user lifespan and overall system lifecycle.

### 3.3. Algorithm Design

#### 3.3.1. SF Transfer Algorithm

Based on the historical records of the satellite gateway, the number of users expected N to be served in each frame can be obtained. The minimum SF distribution $M_{init}$ required to ensure that all users successfully receive the signal is expressed as:

(29) $$M_{init}=\{M_{init,i}|M_{init,i}=P_{i},i=7,8,9,10,11,12\},$$

where $P_{i}$ represents the proportion of SF i.

The optimal distribution of SFs $P_{sf}$ can be obtained from Equation (19). For example, when the minimum SF for successful demodulation in the system is SF7, the optimal distribution is $P_{sf}=\{45.0\%,25.7\%,14.5\%,8.0\%,4.4\%,2.4\%\}$.

The difference $K_{m}$ between the optimal distribution $P_{sf}$ and the minimum SF distribution $M_{init}$ is used to determine whether $M_{init}$ can be transformed into $P_{sf}$.

(30) $$K_{m}=\{m_{i}|m_{i}=M_{init,i}-P_{sf,i},i\in \{7,8,\ldots ,12\}\}.$$

Since the users’ SF can only be adjusted from lower to higher values, it is not possible to use the optimal proportional distribution according to Equation (30). Therefore, an algorithm needs to be designed to determine the new proportional allocation.

(31) $$\exists z,\sum_{l=z}^{12}m_{l}>0,z\in \{8,9,10,11,12\},m_{l}\in K_{m}.$$

Inspired by the work in [5], this paper adds a method for allocating user SF based on transfer probability and proposes an SF proportional allocation algorithm designed to align the distribution of specific SFs with the success rate relationship model. The objective is to minimize data collisions within the IoT system. The dynamic changes in satellite communication links are mainly reflected in the real-time fluctuations of path loss, which can be calculated in real time with high precision through satellite ephemeris data and user geographic location information. Therefore, even when the satellite is in high-speed motion, the fast convergence of the system can still be ensured. The detailed process is outlined in Algorithm 1.

Usually, the minimum SF distribution is derived from historical scheduling records of the satellite gateway. For cold-start cases (e.g., first deployment with no historical data), pre-training is required. The system is trained using scheduling data simulated from typical scenarios and system design parameters. This allows the deployed system to temporarily use pre-trained data as surrogate historical data for calculations, bypassing the lack of real historical records. Next, the optimal scale distribution is determined using Equation (17). Subsequently, an assessment is conducted to determine whether the minimum SF distribution can be transformed into the optimal distribution as per Equation (29).

If transformation is possible, the target SF user distribution is established based on the optimal ratio distribution and the expected number of users to be served. Conversely, if transformation is not feasible, the proportion of users assigned to the first SF—those that cannot be optimally distributed—is arranged according to a suboptimal distribution, while the previous distribution is retained as the optimal ratio distribution. The suboptimal distribution adheres to Equation (15) and is calculated after excluding the probability that guarantees the optimal proportional distribution.

Based on the obtained target SF distribution, the transfer probability of each SF can be derived through the SF transfer algorithm. The reason for using the SF transfer probability here is that in satellite IoT LoRa, the expected number of users is usually large. For a large number of users, it is often difficult to perform simultaneous transfer operations for the SF. Therefore, in this situation, it is common to use the transfer probability method to transfer each SF.

The transfer probability of each SF is determined based on the current number of expected users N in the beacon frame, the minimum SF ratio distribution $M_{init}$, and the target SF user distribution $N_{final}$. The matrix Probability consists of the transfer probabilities corresponding to different SFs and serves as the output. The pseudocode is as follows:
**Algorithm 1**: SF Transfer AlgorithmINPUTNumber of users N, Minimum SF distribution $M_{init}$, Optimal SF distribution $P_{sf}$
OUTPUTProbability**1**Calculate $m_{i}$ and $S_{i}$ from $M_{init}$ and $P_{sf}$
**2**   **For** k = 7 to 12**3**    **If** $S_{k}<$ 0.**4**      *point* = k, $M_{final,point\;}$= $M_{init,point}$
**6**      $M_{final,t}$= $P_{sf,t}$, *t* = 7 to point − 1**8**      $M_{final,t}$= $P_{sf,t}/(1-\sum_{7}^{k}M_{init,i})$, *t* = *point* + 1 to 12**10**
    **else.**
**11**      $M_{final,t}$= $P_{sf,t}$, *t* = 7 to 12**12**
    **End if**
**13**
   **End for**
**14**$N_{final}=\{N_{final,t}|N_{final,t}=N*{\;M}_{final,t}\}$**15**Initialize ${sf}^{original}$ and ${sf}_{i}^{final}$
**16****FOR** 
i=1:N
**17** 
$Numerical\left[{sf}_{i}^{original}-6\right]\left[{sf}_{i}^{final}-6\right]+=1$
**18****End for****19****FOR** i =1:6**20** **FOR** j =1:6**21**  
**If** 
$M_{init}[i]\neq 0$
**22**  
$Probability\left[i\right]\left[j\right]=Numerical\left[i\right]\left[j\right]/{(N*M}_{init}[i])$
**23**  
**Else**
**24**    **If** i ≠ j**25**    
$Probability\left[i\right]\left[j\right]=0$
**26**    
**else**
**27**    
$Probability\left[i\right]\left[j\right]=1$
**28**    
**End if**
**29**  
**End if**
**30** 
**End for**
**31****End for**

Particularly, in scenarios with high real-time data requirements, high priority users are assigned SF7 and SF8 in priority. After the high priority users have received their assignments, other general users will be assigned, and the final overall assignment still satisfies the probability distribution.

#### 3.3.2. Practical Power Control Mechanism

Once the transfer probability is obtained using the aforementioned algorithm, the user employs this probability to determine the SF for the currently transmitted packet. Similar to traditional wireless sensor nodes, satellite IoT LoRa terminals have limited battery life, making low power consumption techniques crucial. To effectively reduce energy consumption, in addition to enabling the node to enter a low-power mode or dormant state when idle, this paper further investigates a power control mechanism based on the previously discussed SF optimization method. This strategy aims to minimize transmission power while maintaining normal communication with the satellite, thereby reducing energy consumption and extending the node’s lifespan.

However, the dynamic nature of satellites leads to continuous changes in the distance between the satellite and the ground terminal, necessitating frequent adjustments to the power control of user terminals. This need for frequent adjustments can significantly increase computational demands. A power control mechanism that relies on constant monitoring of the satellite-ground terminal relationship contradicts the IoT principle of maintaining simple and efficient terminal operations. Therefore, this paper proposes a method akin to adaptive modulation and coding schemes to manage user terminal power within a defined range. The objective is to streamline the power control process while effectively managing energy for ground terminals. At the satellite receiver, the received power is obtained by Equation (1).

The FSPL in Equation (1) is the free space propagation loss, represented as

(32) FSPL=20lg(d)+20lg(f)−147.6,

where d represents the distance between the terminal and the satellite, and f is the carrier frequency.

On the other hand, the sensitivity threshold is obtained by Equation (2).

For successful reception of the packet, the received power needs to be greater than the sensitivity threshold from Equations (2), (3) and (32).

(33) $$p_{i}+20\mathrm{lg}(d)+20\mathrm{lg}(f)-147.6+G_{r}-A_{\mathrm{loss}}-Ad_{\mathrm{loss}}\geq S_{SF}.$$

For the practical implementation of power control mechanisms, the sensitivity threshold table can be referenced as Table 1 [11]:

To simplify the power control process for IoT terminals, this paper utilizes the distance between the satellite’s sub-satellite point and the user terminal for user classification. This method helps avoid complications that may arise from directly using distance for classification. By calculating the maximum distance between the terminal and the satellite within this range, we can determine the minimum transmit power required for the terminal users.

**Figure 4:**
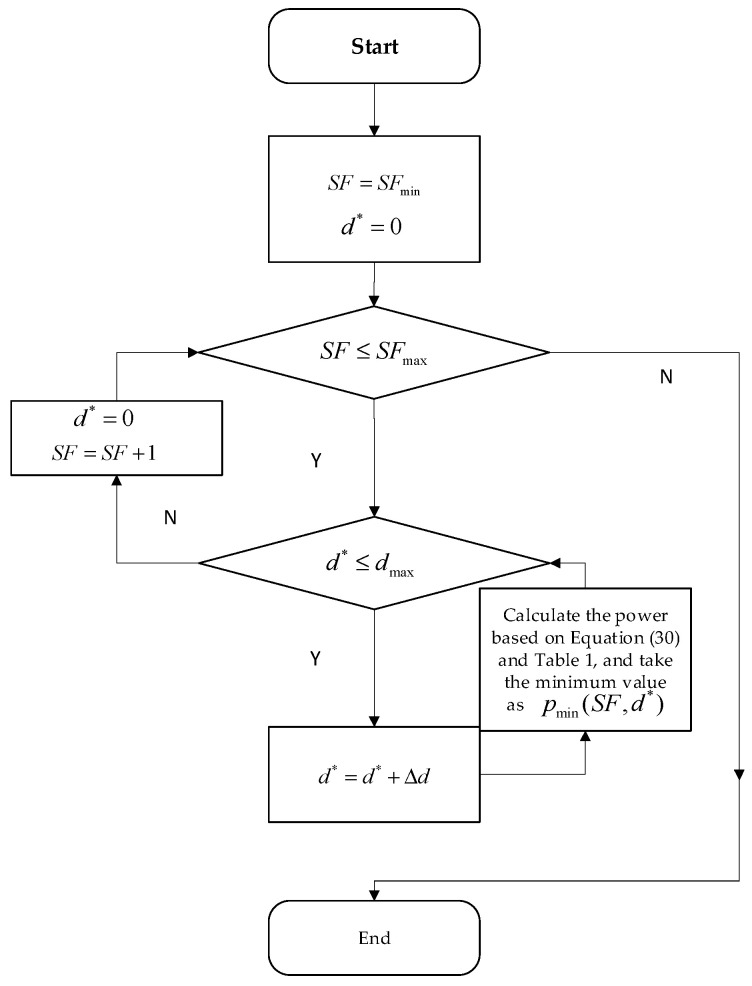
Power control table algorithm.

Users within this range will operate at the calculated power level. In practical applications, the terminal only needs to combine the user’s location information with the ephemeris data to derive the sub-satellite point distance. Utilizing a power control table, the required power can then be easily determined. This power control mechanism not only simplifies the power control process and enables effective power management for users within a defined range, but it also reduces the frequency of power changes for the terminal. The algorithm of power control table is illustrated in Figure 4. In the algorithm, $d^{*}$ is the distance between sub-satellite point and the user, Δd is the distance interval in power control and $p_{i,\min}(SF,d^{*})$ represents the power. For example, the results obtained by taking h=1000km, $d_{\max}=500\mathrm{km}$ and Δd=100km are shown in Table 2.

To ensure that the distance interval for power control can be dynamically adjusted, the following is given according to

(34) $$10\mathrm{lg}\frac{h^{2}+{d_{\max}}^{2}}{h^{2}+{\left(d_{\max}-\Delta d\right)}^{2}}\leq \beta ,$$

where β denotes maximum allowable percentage power difference of the device, h denotes satellite altitude and $d_{\max}$ denotes maximum distance between the sub-satellite point and the user. From the above equation, it is evident that the distance interval for power control is closely associated with both the satellite altitude and the satellite overpass time. This interval can be adaptively adjusted to optimize performance, ensuring that power control strategies remain effective under varying operational conditions. According to Equation (34), Δd can be obtained after h, $d_{\max}$ and β are determined. 

## 4. Simulation and Analysis

### 4.1. Simulation

In this paper, the Simpy-based LoRaSim simulation simulator is used to simulate the communication process of a LoRa-based LEO satellites in order to evaluate the packet extraction under different conditions [24]. The network scenario contains individual satellites as well as IoT users uniformly distributed within a radius of 150 km centered at 34.32 degrees north latitude and 108.55 degrees east longitude. The simulation parameters are shown in Table 3. In the simulation, the details of the probability distributions for stochastic elements are as follows: The packet transmission interval of each user follows an exponential distribution, which is characterized by the packet arrival rate λ. Meanwhile, the location distribution of user nodes adopts a uniform distribution.

This paper compares four resource optimization methods: the min-AirTime and min-AirTime&TP scheduling strategies from the literature [3], the EXPLoRa scheduling strategy from the literature [6], and the Traj.Rnd.Skip scheduling strategy from the literature [11].

The min-AirTime scheduling policy assigns SFs based on the Received Signal Strength Indicator (RSSI) values received by the satellite gateway. Users close to the satellite, indicated by high RSSI values, are assigned lower SFs. However, due to the dynamic nature of the satellite, users tend to stick with the SF determined by RSSI when they first enter the satellite’s line of sight, which limits the utilization of SF orthogonality.

In contrast, the min-AirTime&TP strategy builds upon the min-AirTime approach by optimizing transmit power to save energy. While this does not change the network’s extraction rate, it does improve energy efficiency.

The EXPLoRa scheduling strategy selects SFs for end users based on the number of connected devices within the gateway’s coverage area. In cases where RSSI constraints are present, the SF is distributed equally among all connected end users. This strategy utilizes more SFs, but since packets with different SFs occupy varying amounts of channel time, an appropriate allocation of SF ratios is necessary to maximize packet extraction. Additionally, due to the dynamic nature of the satellite, users may be assigned lower SFs when they are farther away, resulting in discarded packets that cannot be demodulated.

The Traj.Rnd.Skip scheduling strategy, also known as the trajectory random hopping strategy, employs a beacon-based data frame structure model that takes into account the satellite’s dynamics. However, it relies solely on trajectory information to randomly select a SF based on the minimum SF capable of demodulation. This approach does not fully utilize SF orthogonality or consider the variable power characteristics of LoRa terminals, leading to energy inefficiency.

The algorithm proposed in this paper addresses the movement of LEO satellites and demonstrates advantages in terms of both energy efficiency and data extraction rate compared to the four aforementioned strategies.

### 4.2. Performance Analysis and Discussion

To simulate the large-scale deployment of IoT terminals, the number of terminals in the experiment is N∈{100,200,300,500,750,1000,1250,1500,1750,2000,2250,2500,2750,3000}.

**Figure 5:**
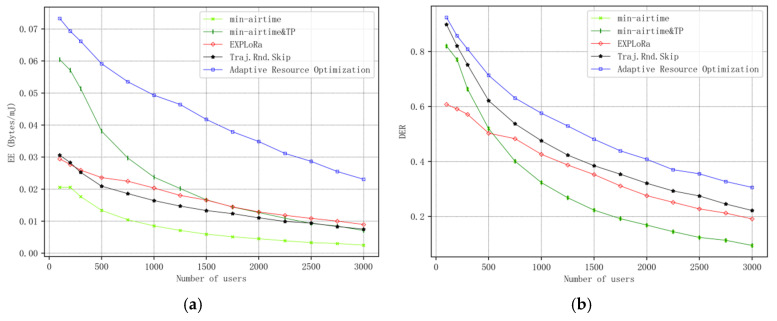
(**a**) Curve of energy efficiency EE with number of users. (**b**) Curve of data extraction rate DER with the number of users.

Figure 5a illustrates the curve of system energy efficiency (EE) as a function of the number of users, while Figure 5b shows the curve of data extraction rate (DER) in the network as the number of users varies. It can be observed that the proposed algorithm demonstrates significant improvements in both EE and DER compared to existing algorithms in the literature, with the enhancement in EE being more pronounced than that in DER. This indicates that the proposed algorithm performs better in optimizing energy consumption and data extraction.

In terms of algorithm complexity, by analyzing the algorithm presented in this paper, the algorithm complexity can be further calculated and compared with other algorithms. The algorithm in this paper only performs iterations over the number of users N and the six fixed types of SF. Since N is much larger than SF, the complexity is O(N). The other algorithms primarily iterate over the number of users as well, resulting in a complexity of O(N). Thus, the complexity of the algorithm in this paper is comparable to that of other algorithms. However, the performance of the algorithm presented in this paper is superior to that of other algorithms.

**Figure 6:**
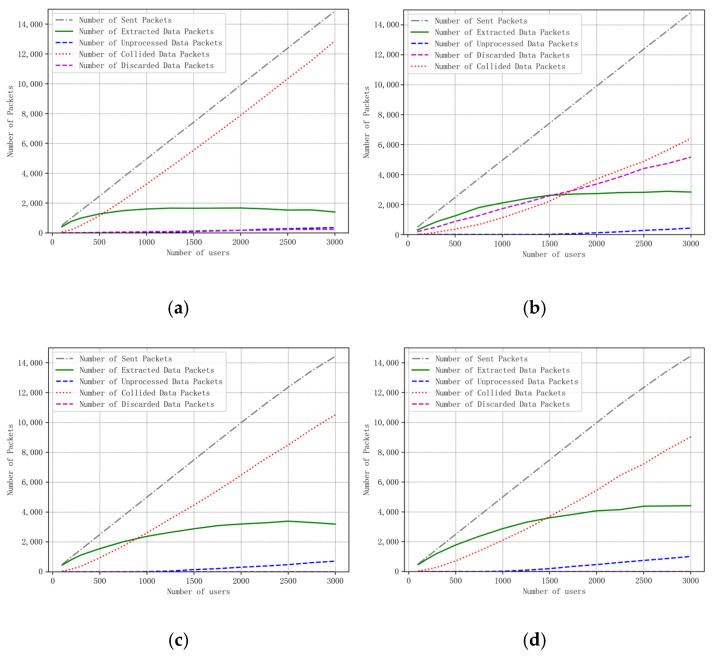
Curve of packet state in the network with the number of users. (**a**) min-airtime. (**b**) EXPLoRa. (**c**) Traj.Rnd.Skip. (**d**) Algorithm of this paper.

Figure 6 illustrates the curve of packet status in the network as the number of users varies. It can be observed that, compared to existing algorithms in the literature, the proposed algorithm reduces the number of collisions and discarded packets while increasing the number of successfully extracted packets.

In the min-AirTime strategy, users always select the SF determined by the Received Signal Strength Indicator (RSSI) when they first enter the satellite’s line of sight. This conservative choice of SF results in an increased number of collisions and a decrease in the successful extraction of packets within the system. For the EXPLoRa mechanism, users consistently adopt an evenly distributed SF, which means that, regardless of whether it is at the beginning or during subsequent transmissions, some users’ packets will be discarded due to not meeting demodulation conditions. The Traj.Rnd.Skip algorithm adopts a beacon-triggered data frame model to divide the satellite pass time into several frames and uses a jump parameter to make data packets spread almost throughout the entire satellite pass time [11]. Additionally, during the middle period of the satellite pass, when the user is exactly under the satellite and the distance to the satellite is relatively short, the Traj.Rnd.Skip algorithm randomly selects higher spreading factors based on the minimum spreading factor for successful demodulation to ensure demodulation. The difference between the algorithm proposed in this paper and the Traj.Rnd.Skip algorithm lies in that it determines the optimal or suboptimal proportion distribution based on the success rate relationship model, fully utilizes the orthogonality of spreading factors, and improves the amount of successfully extracted data in the system.

**Figure 7:**
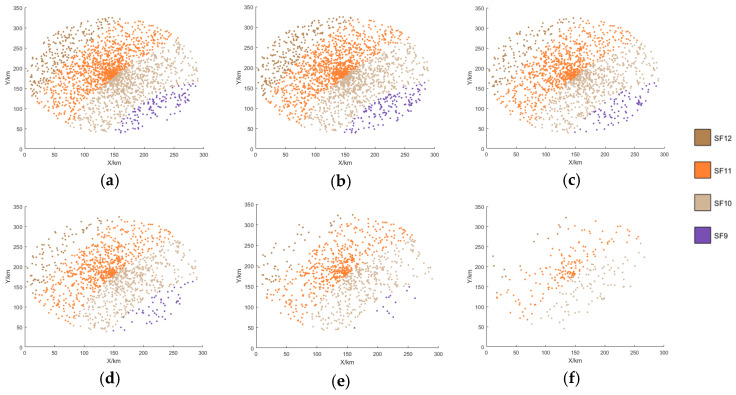
SF distribution of the min-AirTime algorithm. (**a**) 0–60 s SF distribution. (**b**) 60–120 s SF distribution. (**c**) 120–180 s SF distribution. (**d**) 180–240 s SF distribution. (**e**) 240–300 s SF distribution. (**f**) 300–360 s SF distribution.

**Figure 8:**
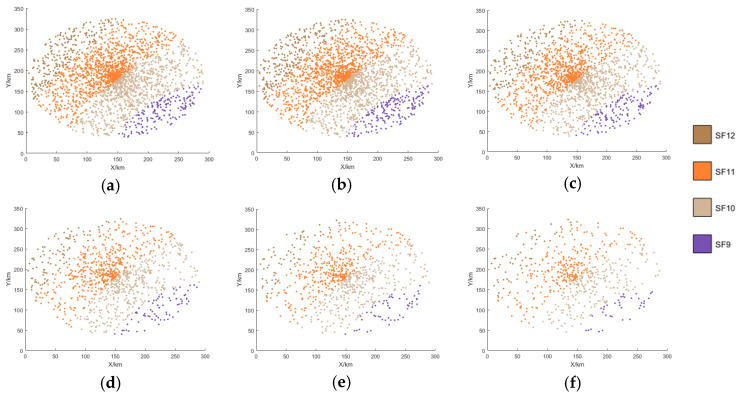
SF distribution of the EXPLoRa algorithm. (**a**) 0–60 s SF distribution. (**b**) 60–120 s SF distribution. (**c**) 120–180 s SF distribution. (**d**) 180–240 s SF distribution. (**e**) 240–300 s SF distribution. (**f**) 300–360 s SF distribution.

**Figure 9:**
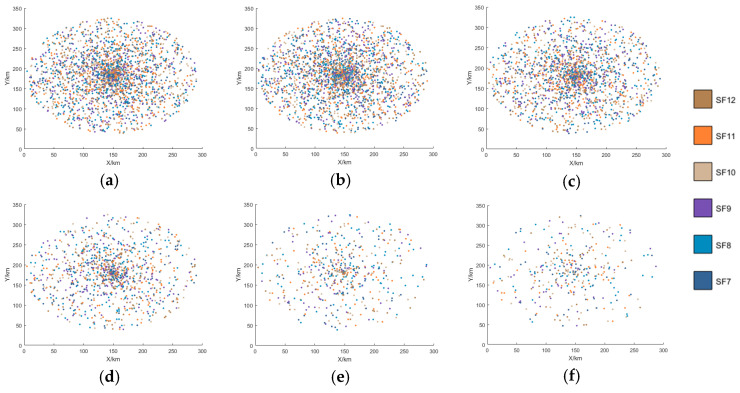
SF distribution of the Traj.Rnd.Skip algorithm. (**a**) 0–60 s SF distribution. (**b**) 60–120 s SF distribution. (**c**) 120–180 s SF distribution. (**d**) 180–240 s SF distribution. (**e**) 240–300 s SF distribution. (**f**) 300–360 s SF distribution.

**Figure 10:**
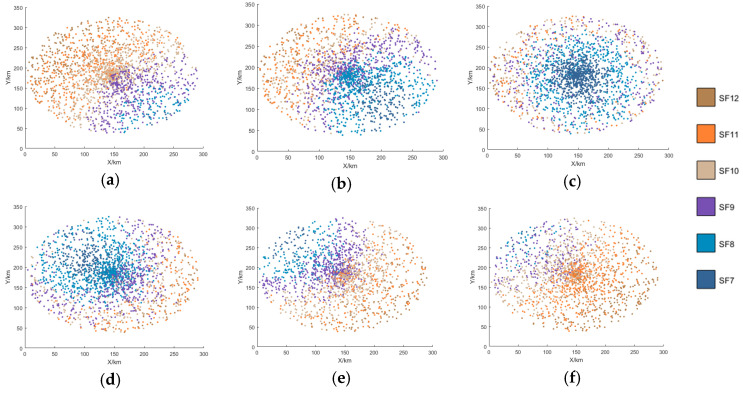
SF distribution of this paper’s algorithm. (**a**) 0–60 s SF distribution. (**b**) 60–120 s SF distribution. (**c**) 120–180 s SF distribution. (**d**) 180–240 s SF distribution. (**e**) 240–300 s SF distribution. (**f**) 300–360 s SF distribution.

**Figure 11:**
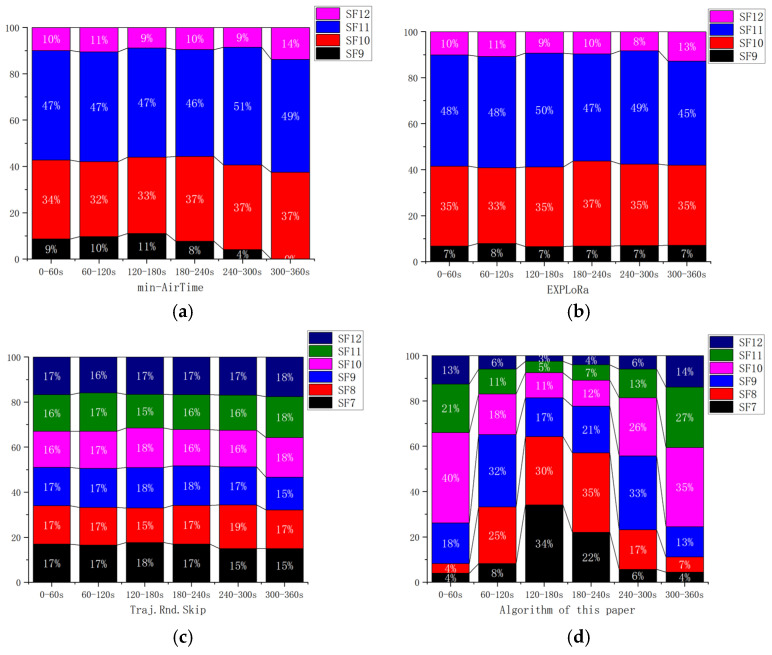
Stacked column charts based on the results of the Monte-Carlo simulations. (**a**) min-airtime. (**b**) EXPLoRa. (**c**) Traj.Rnd.Skip. (**d**) Algorithm of this paper.

Figure 7, Figure 8, Figure 9 and Figure 10 display the distribution of SFs corresponding to the data packets sent by users during the satellite’s passage. This paper conducts 100 Monte-Carlo simulations for the random distribution of SF. Based on the results of the Monte-Carlo simulations, stacked column charts are shown in Figure 11.

Since the min-AirTime algorithm performs SF allocation based on users’ location distribution and the RSSI, the algorithm exhibits a conservative selection of SFs, only adopting SF9-SF12 without fully utilizing all available SFs. Additionally, in the final time period, SF9 is not used at all, as shown in Figure 7 and Figure 11a. This indicates that the min-AirTime algorithm is overly conservative in SF allocation, leading to uneven SF distribution. Furthermore, the algorithm has certain issues in temporal allocation, characterized by excessive allocation in the early stage of satellite pass and insufficient allocation in the late stage.

The EXPLoRa algorithm adopts a uniform SF allocation strategy across all connected users, resulting in basically unchanged SF distribution during the satellite’s dynamic movement, as shown in Figure 8 and Figure 11b. However, due to resource limitations, the algorithm can only use SF9-SF12, failing to fully utilize all SFs. Compared with the min-AirTime algorithm, EXPLoRa has a relatively more uniform temporal distribution, but its distribution still cannot meet the dynamic movement characteristics of the satellite.

**Figure 12:**
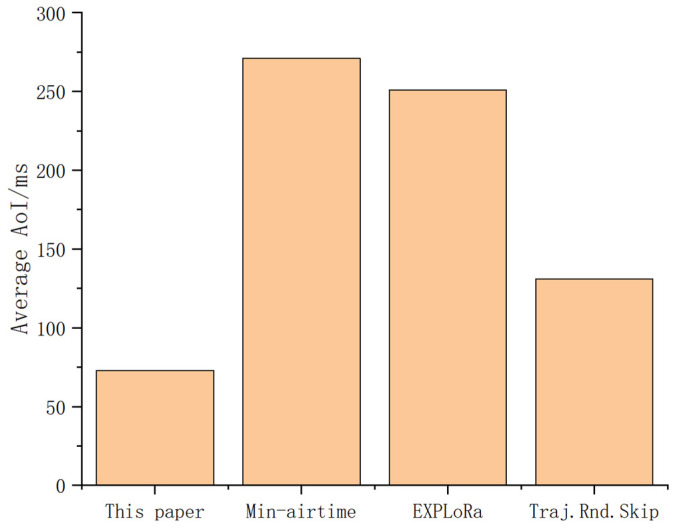
Average AoI of different algorithms.

The Traj.Rnd.Skip algorithm employs a beacon-triggered data frame model that divides the satellite’s passage time into several frames. It also uses hopping parameters to spread data packets as evenly as possible throughout the satellite’s passage. However, the Traj.Rnd.Skip algorithm does not account for optimal ratio distribution, leading to sparse packet distribution in later time periods, as shown in Figure 9 and Figure 12c. Since the Traj.Rnd.Skip algorithm relies on trajectory information to randomly select an SF based on the minimum SF capable of demodulation, it results in a relatively uniform distribution in SF allocation.

The algorithm presented in this paper represents a significant leap forward from the Traj.Rnd.Skip algorithm, leveraging a success rate relationship model to meticulously determine the optimal ratio distribution. In terms of temporal distribution, it outperforms previous algorithms by ensuring an evenly balanced packet transmission throughout the satellite’s passage, effectively resolving the issue of sparse packet distribution in later time periods that plagued other methods, as shown in Figure 10 and Figure 11d.

When it comes to SF allocation, the algorithm excels at adapting to the satellite’s dynamic movement. It does not merely follow the satellite’s trajectory but actively adjusts the SF distribution in real-time to match the changing communication conditions during the satellite’s movement. This dynamic adaptability in SF allocation, combined with its superior temporal distribution capabilities, enables the algorithm to consider the periodic changes in SF distribution corresponding to the satellite’s cyclical coverage—a feature that remains beyond the reach of existing algorithms.

In scenarios with high real-time data requirements, high priority users are assigned SF7 and 8 in priority. Figure 12 shows the average AoI values of different algorithms, among which the algorithm proposed in this paper achieves the lowest AoI.

## 5. Future Work

It should be noted that the dynamic resource optimization algorithm proposed in this paper still has certain limitations. The research mainly focuses on resource optimization in single-satellite scenarios and does not consider the impact of inter-satellite handover processes in multi-satellite constellation scenarios on SF allocation and power control strategies. During inter-satellite handover, changes in signal strength, interference in overlapping coverage areas, and differences in channel characteristics between satellites may cause the existing SF allocation scheme to fail to effectively guarantee system energy efficiency and data transmission stability. Additionally, this paper does not conduct in-depth discussions on the dynamic power adjustment mechanism during handover, which are all urgent directions to be improved.

**Figure 13:**
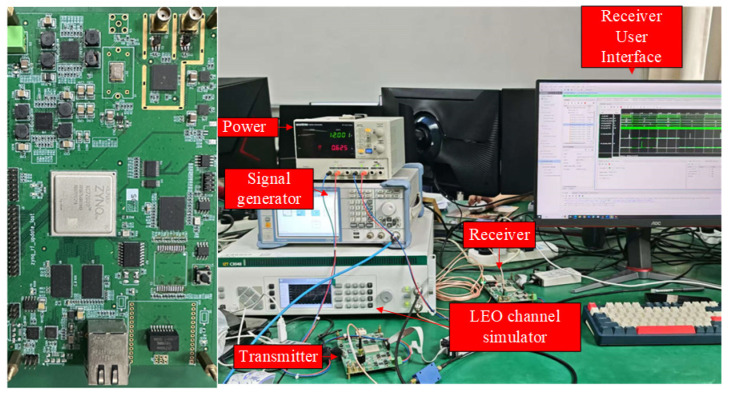
Testbed for hardware validation tests.

In future work, the following aspects will be explored:

1. The proposed dynamic resource optimization algorithm will be extended to multi-satellite constellation scenarios. In these scenarios, inter-satellite handover and coordinated allocation of SFs in overlapping coverage areas will pose new challenges, while also providing opportunities for further optimizing energy efficiency.
2. Machine learning techniques, such as deep reinforcement learning, will be integrated to enable the algorithm to adapt to more complex and unpredictable channel conditions.
3. To conduct hardware validation tests, a testbed has been set up in the laboratory, including power supplies, signal generators, LEO channel simulators, etc. Opportunities for conducting field tests will be sought in the future.The testbed for hardware validation tests is shown in Figure 13.

## 6. Conclusions

In this paper, a dynamically optimized resource optimization algorithm is proposed for the resource optimization problem in LoRa LEO satellite IoT scenarios, aiming to maximize the energy efficiency of the system and the data extraction rate of the system. The algorithm can dynamically allocate the SF as the satellite moves, while using a success rate relationship model to ensure that the orthogonality of the SF is fully utilized.

Simulation results demonstrate that the resource optimization algorithm proposed in this paper significantly outperforms existing schemes in system energy efficiency and data extraction rate. Specifically, when the number of users reaches 3000, the energy efficiency is improved by at least 119%, and the data extraction rate is increased by at least 48%, highlighting the algorithm’s effectiveness in optimizing resource utilization for IoT.

## Figures and Tables

**Table 1:** Table of sensitivity thresholds.

SF	Sensitivity Threshold/dBm		
	BW=125 KHz	BW=250 KHz	BW=500 KHz
7	−123	−120	−117
8	−126	−123	−120
9	−129	−126	−123
10	−132	−129	−126
11	−134.5	−131.5	−128.5
12	−137	−134	−131

**Table 2:** Table of power.

SF	$p_{i,\min}(SF,d^{*})/\mathrm{dBm}$				
	$d^{*}\in [0,100)$	$d^{*}\in [100,200)$	$d^{*}\in [200,300)$	$d^{*}\in [300,400)$	$d^{*}\in [400,500)$
7	17.71	17.84	18.04	18.31	18.64
8	14.71	14.84	15.04	15.31	15.64
9	11.71	11.84	12.04	12.31	12.64
10	8.71	8.84	9.04	9.31	9.64
11	6.21	6.34	6.54	6.81	7.14
12	3.71	3.84	4.04	4.31	4.64

**Table 3:** Simulation parameters.

Parameters	Meaning	Value
$h_{s}/\mathrm{km}$	LEO Satellite Altitude	1000
N	Number of users	[100, 3000]
$p_{i}/\mathrm{dBm}$	power	[3, 20]
PL/Byte	Packet payload length	20
m	Number of cached packets	5
C	channel count	3
$p_{skip}$	skip parameter	4000
SF	SF	7–12
BW/KHz	bandwidth	125
$T_{f}/s$	frame duration	30
$T_{p}/s$	Satellite transit time	360
f/MHz	carrier frequency	868
L	Number of packets processed simultaneously by the satellite	16
$G_{r}/\mathrm{dbm}$	Antenna Gain	12
$A_{\mathrm{loss}}/\mathrm{dbm}$	Atmospheric losses due to gases, rain, etc.	0.5
$Ad_{\mathrm{loss}}/\mathrm{dbm}$	Additional losses due to feedline	1

## Data Availability

The original contributions presented in this study are included in the article. Further inquiries can be directed to the corresponding author.
